# Reactions to threatening health messages

**DOI:** 10.1186/1471-2458-12-1011

**Published:** 2012-11-21

**Authors:** Gill A ten Hoor, Gjalt-Jorn Y Peters, Janice Kalagi, Lianne de Groot, Karlijne Grootjans, Alexander Huschens, Constanze Köhninger, Lizan Kölgen, Isabelle Pelssers, Toby Schütt, Sophia Thomas, Robert AC Ruiter, Gerjo Kok

**Affiliations:** 1 Dept. of Work & Social Psychology, Faculty of Psychology & Neuroscience, Maastricht University, P.O. Box 616, 6200, MD, Maastricht, The Netherlands; 2 Dept. of Research Methods & Statistics, Faculty of Psychology, Open University, P.O. Box 2960, 6401, DL, Heerlen, The Netherlands

**Keywords:** Threatening health messages, Defensive reactions, Smokers, Drinkers

## Abstract

**Background:**

Threatening health messages that focus on severity are popular, but frequently have no effect or even a counterproductive effect on behavior change. This paradox (i.e. wide application despite low effectiveness) may be partly explained by the intuitive appeal of threatening communication: it may be hard to predict the defensive reactions occurring in response to fear appeals. We examine this hypothesis by using two studies by Brown and colleagues, which provide evidence that threatening health messages in the form of distressing imagery in anti-smoking and anti-alcohol campaigns cause defensive reactions.

**Methods:**

We simulated both Brown et al. experiments, asking participants to estimate the reactions of the original study subjects to the threatening health information (n = 93). Afterwards, we presented the actual original study outcomes. One week later, we assessed whether this knowledge of the actual study outcomes helped participants to more successfully estimate the effectiveness of the threatening health information (n = 72).

**Results:**

Results showed that participants were initially convinced of the effectiveness of threatening health messages and were unable to anticipate the defensive reactions that in fact occurred. Furthermore, these estimates did not improve after participants had been explained the dynamics of threatening communication as well as what the effects of the threatening communication had been in reality.

**Conclusions:**

These findings are consistent with the hypothesis that the effectiveness of threatening health messages is intuitively appealing. What is more, providing empirical evidence against the use of threatening health messages has very little effect on this intuitive appeal.

## Background

Threatening health messages that focus on severity in an emotional way are often used as a strategy to reduce unhealthy behaviors [1,2]. However, people rarely change their behavior as a result of threatening health messages [3]. Generally, threatening health messages frequently result in defensive reactions [4-6], (Van 't Riet J, Ruiter RAC, Defensive reactions to health-promoting information: An overview and implications for future research. Submitted.) which cause them to have no effect [3,7], or, on occasion, to have a counterproductive effect [3]. Brain imaging techniques have confirmed that people who are most at risk pay the least attention to threatening messages [8,9]. Reviews of the limited effectiveness of threatening health messages have been available for over ten years [3,10-15], obviously without much impact on the attractiveness of using it in behavior change interventions. The most recent meta-analysis resolved a number of problems of previous meta-analyses, finding clear evidence for a significant interaction between threat and efficacy such that threat only had an effect under high efficacy and efficacy only had an effect under high threat [3]. This meta-analysis also showed that under low efficacy the effect of threat was negative and almost significant, which means that if an intervention developer is not very certain that either the target population is high in response and self-efficacy, or that a given intervention will manage to considerably increase both response and self-efficacy, threatening messages should be avoided.

Brown and colleagues have shown that distressing messages that emphasize the severity of a health threat cause defensive reactions. In two studies, the subjects (smokers [16] and regular alcohol consumers [17]) were presented with booklets containing either highly distressing pictures or less distressing pictures. After reading these booklets, the participants rated their own risk and their perceived effectiveness of the booklets. Results of the smoking study [16] showed that rather than having increased their risk perceptions, participants who read distressing booklets reported lowered risk perceptions and evaluated the messages more negatively than participants in the less distressing condition. Results of the alcohol study [17] showed similar effects, although here the defensive reactions were only significant in those regular drinkers who reported both a greater dispositional denial (as measured by the COPE denial scale [18]) and more alcohol-related problems (as measured by a five-item version of the Alcohol Use Disorders Identification Test, AUDIT [19]).

Despite the fact that threatening health messages lead to defensive reactions among those most in need of change, the use of these messages remains well-accepted, even among health professionals [1,2]. So far, limited empirical evidence is available as to why severity-based threatening health messages remain popular despite the distribution of information regarding their ineffectiveness, and as to what measures can be taken to reduce this popularity. In this study, first, we assessed participants’ knowledge of the effectiveness of threatening health messages, in particular whether or not they would expect threatening health information to cause defensive reactions. Second, we examined whether presenting participants with information about the actual effects that were found in the Brown et al. studies would change their estimates of the effectiveness of threatening health messages in behavior change programs. To study these research questions, we first copied the materials from the two studies by Brown and colleagues to assess whether participants (students, as in the Brown et al. studies) were able to predict the defensive reactions found in these studies. Then, regardless of their answers, we informed the students about the actual outcomes of the two simulated studies. One week later, participants were again asked to estimate the effectiveness of a threatening message, albeit for a message about a different behavior. Based on the popularity of threatening health messages, we hypothesized that participants expect threatening health information to be effective and that they are unable to predict defensive reactions. In addition, we hypothesized that an intervention that provides empirical evidence against the use of threatening health information can curb the perceived effectiveness of threatening health messages.

## Methods

### Full disclosure

Following recent pleas for full disclosure of research materials, data, analyses and output [20,21], the questionnaires (in LimeSurvey XML survey format, .lss), stimulus materials (in Portable Document Format, .pdf), and the intervention (in the LimeSurvey files) are combined in a .zip archive as Additional file 1 and in a scientific repository at http://sciencerep.org/3/.

### Participants

**Figure 1:**
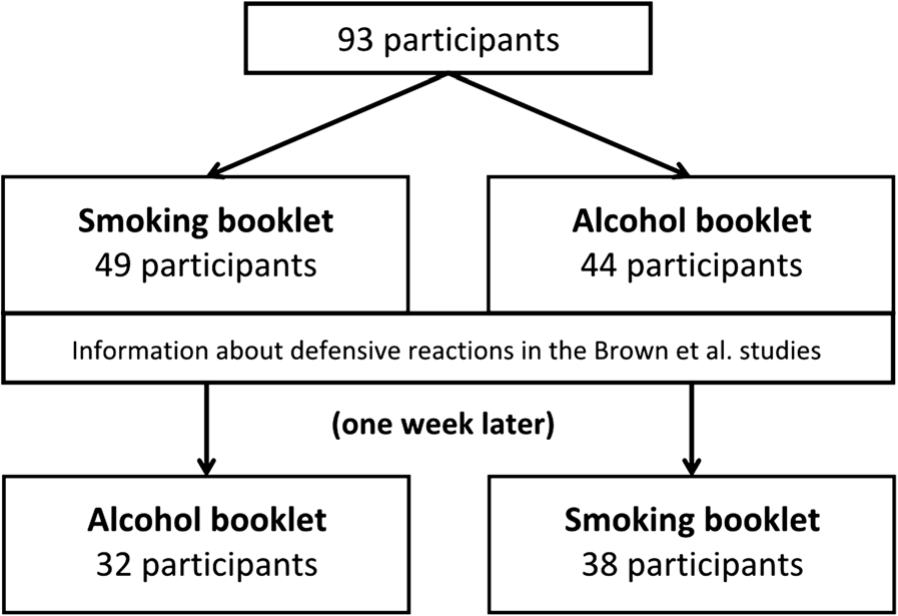
Flowchart illustrating the procedure of the study and the number of participants.

In total, 134 university students (18 to 29 years of age) enrolled in different curricula at different universities were approached through personal contact with the request to participate in an experiment about threatening health messages. Psychology students were excluded, as it was likely they had already been exposed to the information we would provide in the study. Of the 134 invited participants, 93 participants completed the first part of the study (t_0;_ see Figure 1), with a mean age of, 21.63 years (median 21, standard deviation 2.1), of whom 52 were female. Of these, 44 participants read the alcohol booklets (see the Procedure section) and 49 participants read the smoking booklets. Most participants (n = 71) completed the second part of the study (t_1_), where 32 rated the alcohol booklets and 38 rated the alcohol booklets (see Procedure section). Among the 71 participants who completed both parts of the study, three € 50 gift vouchers were raffled. The experimental materials and procedure were approved by the Ethics Committee of the Faculty of Psychology and Neuroscience, Maastricht University.

### Procedure

Upon agreeing to participate, participants were asked for their e-mail address after which they received a personalized link to the first part of the online experiment. The experiment was presented using the open source package LimeSurvey. The link opened an instance of LimeSurvey [22] where participants were asked to participate in two tests (t_0_ and t_1_) with an intervention in between (see Figure 1). Subsequently, participants gave their informed consent by clicking on the appropriate button and were randomized into one of two groups. Participants in the first group were presented with the two booklets about smoking, whereas participants in the other group were presented with the two booklets about drinking. In both groups, the participants read both one booklet with highly threatening images (HT) and one with less threatening images (LT). At the start of t_0_, the participants were first informed about the procedures of the original experiment (either the smoking or the drinking study, according to a participant's group), but they were given no information about the results. Then, participants were informed about the characteristics of the subjects of the original experiments (either people who smoke at least one cigarette per day or people who drink at least six glasses of alcohol at least twice a week) and instructed to estimate how the original subjects rated the booklets. During the entire test, participants were able to switch between the LT and HT booklets. After the participants read the booklets, their gender and age were measured, followed by questions assessing their predictions as to how the subjects in the original experiments [16,17] evaluated the health messages and how they responded to questions about personal risk and emotional impact. Finally, participants’ own smoking and drinking behaviors were measured.

Immediately after t_0_, participants were presented with an intervention consisting of information about the actual defensive reactions to threatening health messages in the Brown et al. study and an explanation of the dynamics of processing threatening information (see Intervention section). One week after t_0_, t_1_ followed. Participants were asked not to talk about the study in between t_0_ and t_1_. At t_1_, participants who read the booklets about smoking during t_0_ now read the booklets about alcohol and vice versa, to make sure that we measured understanding of the dynamics of threatening messages on a general level, instead of simple recognition. After participants inspected the booklets, the same measures were completed as at t_0_. At the end of the study, participants were debriefed.

### Stimulus materials

To maximize accuracy of our simulation, the materials (booklets and questionnaires) from the original experiments [16,17] were copied as accurately as possible. However, there were two differences in the procedure: 1) our participants were asked to rate both booklets (high threat messages and low threat messages) instead of a single booklet (see Measures section) and 2) our study was online. Furthermore, our participants used 7-point Likert scales (−3 to 3) for all questions, where in the Brown et al. studies, a variety of scales was used. With permission from Brown, two pairs of booklets were copied from the original studies [16,17]. The booklets provided the same information about the negative consequences of smoking or drinking, but contained different images. In the HT booklets, the pictures were highly distressing. In the smoking booklet, pictures presented a smokers lung; surgically removed cancer of the lower jaw; a gangrenous foot; and lung emphysema. In the alcohol booklet, pictures were presented of an enlarged liver; mouth and throat cancer; chronic pancreatitis; and a fatal car accident. In the LT booklets, less distressing pictures were shown. The smoking booklet contained pictures of cigarettes with the text “smoking causes cancer in a lot of places (including the butt)”; cigarettes in a beer glass with the text “avoid drinking with killers”; a cigarette in the shape of an injection needle with the text “cigarettes kill 17,500 more Australians each year than heroin”; and a picture of cigarettes in the shape of a hand with the middle finger up “The signal from tobacco companies is loud and clear”. The alcohol booklet contained a picture of a fallen glass (‘spilling beer’); a picture of a man drinking beer (‘overuse of alcohol has a range of health effects’); a picture of a toast with beer (‘drinking in rounds can increase consumption’); pictures of cars (‘drink driving can have fatal consequences’), and an advertisement against drink driving. Participants could switch between the original English booklets and Dutch translations.

### Measures

**Figure 2:**
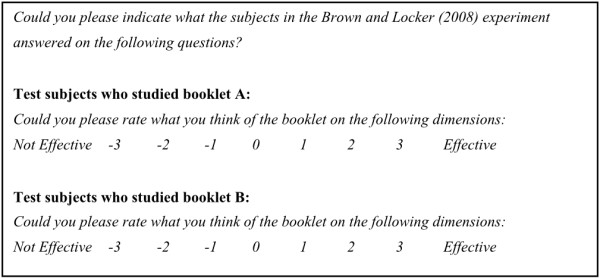
Example questions of how participants rated the booklets.

The measures were replicated from the original studies [16,17]. Each participant provided ratings for both the HT and LT booklets (see Figure 2). Participants were told that it was not important whether they smoked cigarettes or drank alcohol or not, and they were explicitly asked to identify themselves with the smoking or drinking subjects in the original experiments, rather than answering the questions for themselves. As a manipulation check, participants were asked to rate the estimated emotional impact of the booklets. Scores on items that measured the same construct were averaged into one scale. The internal consistency of these scales was good (ω_total_ ranged from .81 to .98; the Greatest Lower Bound, GLB, ranged from .84 to .99; we computed these measures rather than Cronbach's α because of the severe problems of the latter, see e.g. [23,24]). The interested reader can examine the Cronbach's alpha's in our output file in Additional file 1 or at http://sciencerep.org/3/. All questions were formulated as depicted in Figure 2.

#### Emotional impact

Participants estimated the emotional impact that the materials had on the participants in the original studies by answering three items that asked to what extent participants in the original study reported feeling distressed, anxious and uneasy. These items used 7-point Likert scales ranging from −3 (extremely) to 3 (not at all).

#### Evaluation of the booklets

Eight 7-point semantic differentials (ranging from −3 to 3) measured the extent to which participants thought that the original subjects rated the booklets on the following dimensions: *persuasive–not persuasive*, *bad–good*, *clever–stupid*, *not effective–effective*, *absolutely makes me want to quit smoking/decrease drinking–absolutely does not make me want to quit smoking/decrease drinking*, *no weaknesses–significant weaknesses*, *no strengths–significant strengths*, *very important message–unimportant message.* Item scores were recoded and averaged such that higher scores on the aggregate scale reflected higher estimated booklet effectiveness.

#### Risk perception

Our participants estimated original study participants' perceived likelihoods of getting a tobacco or alcohol related disease or problem using six items for smoking and eight items for alcohol consumption. The tobacco related diseases or problems (heart attack, bronchitis, lunch cancer, stroke, cancer of the throat or mouth, emphysema) and alcohol related diseases or problems (serious difficulties in an intimate relationship, become an addict, serious liver disease, the degree to which alcohol makes one insult or be rude to people, trouble with family relationships, serious injuries, conflicts or difficulties with friends, and withdrawal symptoms) were all rated on a 7-point Likert scale, ranging from −3 *(*no chance) to 3 (completely certain), where higher scores reflected a higher estimated risk perception.

Finally, participants were asked about their own smoking and drinking behavior. Smoking behavior was assessed by questions about the smoking status (yes/no), how many cigarettes are smoked during the week and weekend, and the number of previous quit attempts. Alcohol drinking behavior was measured using the AUDIT [19].

### Intervention

The intervention was designed to teach people about the potential negative effects of threatening health messages by providing understandable scientific evidence and explaining why threatening messages are ineffective. The information included a brief explanation of the experimental procedure and outcomes of the Brown et al. studies, showing that fear arousing messages lead to defensive reactions that render them ineffective. In addition, the intervention explained how threatening information is processed. As we estimated that the prevalent model dealing with threatening communication (the Extended Parallel Process Model; [25]) would be conceptually too complex for our participants, we explained the dynamics of threatening communication using cognitive dissonance reduction. Specifically, we explained that people experience cognitive dissonance in situations in which they feel a disharmony between their behavior and beliefs, and that to reduce this disharmony, defensive mechanisms (such as changing beliefs regarding susceptibility to a risk) are used when one’s self-efficacy regarding changing the relevant behavior is low [7]. The message suggested that it would be more effective to motivate people to reduce risk behavior by telling them *how* they can change their unhealthy behavior, instead of scaring them.

### Data analysis

Analyses were conducted using R [26]. In acknowledgement of recent concerns regarding lack of disclosure in scientific research [27], and to aid future meta-analyses, all data, R scripts, and output files are available at http://sciencerep.org/3/ and in the Additional file 1 archive. We report 80% confidence intervals of effect sizes (following Cohen's recommendation; [28]) rather than Null Hypothesis Significance Testing (NHST) outcomes, based on the problems of NHST (e.g. [29]) and pleas to remedy this problematic approach (e.g. [30,31]). However, interested readers can always view the NHST statistics (t values, degrees of freedom, and p-values) in the output file that is available as a text-file at http://sciencerep.org/3/ and in Additional file 1. Because not all readers may be used to working with confidence intervals yet, we will also report whether associations are significant or not; corresponding values for t and the degrees of freedom can be found in the text-file with the original output at http://sciencerep.org/3/ and in Additional file 1.

We first conducted manipulation checks, comparing the estimated emotional impact for the LT and HT booklets. Then, difference scores for the LT and HT ratings for the evaluation and risk perceptions estimates were computed, and potential covariates were explored by examining the bivariate associations of age, sex and behavior (smoking and alcohol use) with the difference scores of evaluation and risk perception estimates (no covariates were associated to these difference scores; see the output file). To test the first hypothesis (LT booklets are rated lower than HT booklets), we computed confidence intervals for Cohen's d for the differences in ratings between the LT and the HT booklets at t_0_. Then, to test the second hypothesis, we computed confidence intervals for Cohen's d for the change in mean HT-LT rating differences between t_0_ and t_1_. In these analyses, we used the pooled standard deviation if variances were equal, and the standard deviation from the largest of the two samples if they were not (see our analysis script at http://sciencerep.org/3/ or in Additional file 1).

## Results

**Figure 3:**
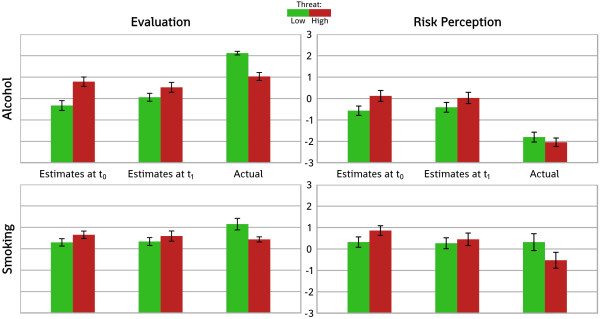
**The mean ratings for estimated risk perception and evaluation, compared to the actual outcomes in the Brown et al. studies.** The error bars indicate 95% confidence intervals around the means.

Participants' ratings of the LT and HT booklets at t_0_ and t_1_, and the differences between the LT and HT ratings, are shown in Table 1 and Figure 3.

**Table 1:** Means and standard deviations of estimated emotional impact, estimated evaluation, and estimated risk perception for the LT and HT booklets in the current study, and the means and standard deviations found in the original studies of Brown et al

			**LT booklet mean (sd)**	**HT booklet mean (sd)**	**LT booklet B&S/B&L mean (sd)**	**HT booklet B&S/B&L mean (sd)**
**t**_**0**_	**Alcohol**	Emotional impact	−1.18 (1.33)	1.20 (1.32)		
	(n=44)	Evaluation	−0.33 (1.12)	0.79 (1.07)	0.26 (0.67)	0.53 (0.38)
		Risk perception	−0.57 (1.07)	0.12 (1.25)	−2.04 (0.71)	−1.8 (0.83)
	**Smoking**	Emotional impact	−0.79 (1.27)	1.10 (1.48)		
	(n=49)	Evaluation	0.30 (0.85)	0.65 (0.86)	0.44 (1.17)	1.15 (0.99)
		Risk perception	0.32 (1.18)	0.86 (1.11)	−0.52 (1.34)	0.22 (1.45)
**t**_**1**_	**Alcohol**	Emotional impact	−0.91 (1.22)	0.81 (1.28)		
	(n=32)	Evaluation	0.06 (0.91)	0.52 (1.13)	0.26 (0.67)	0.53 (0.38)
		Risk perception	−0.41 (1.13)	0.03 (1.29)	−2.04 (0.71)	−1.8 (0.83)
	**Smoking**	Emotional impact	−0.25 (1.24)	1.03 (1.49)		
	(n=38)	Evaluation	0.34 (0.91)	0.59 (1.17)	0.44 (1.17)	1.15 (0.99)
		Risk perception	0.27 (1.26)	0.45 (1.44)	0.52 (1.34)	0.32 (1.45)

Note: participants who evaluated the alcohol booklet at t_0_, evaluated the smoking booklet at t_1_ and vice versa. To enable comparison, scores from the Brown et al. studies were recoded to the metric used in the current study (i.e. ranging from −3 to 3).

### Manipulation checks

Participants accurately predicted that participants experienced the HT booklets as more distressing than the LT booklets, with estimated emotional distress differences of around one standard deviation at t_0_ for both behaviors and at t_1_ for alcohol (Cohen's *d* confidence intervals ranging from 0.86 to 1.76), but a difference of only a fifth to a ninetieth standard deviation for alcohol at t_1_ (Cohen's *d* 0.17-0.93). Null Hypothesis Significance Testing (NHTS) revealed *p*-values of .002 or smaller.

### Differences in estimated ratings (t_0_)

#### Estimated evaluation

Our participants estimated that the participants in the original studies rated the HT booklet higher. For alcohol, the HT booklet was rated especially high, around one standard deviation higher (Cohen's *d* 0.72-1.30); and for smoking around half a standard deviation (Cohen's d 0.15-0.67). NHST revealed respective *p*-values of < .001 and .044.

#### Estimated risk perception

Again, our participants estimated that the participants in the original studies rated the HT booklet higher. The HT booklet was rated around half a standard deviation higher for both alcohol (Cohen's *d* 0.31-0.87) and smoking (Cohen's d 0.21-0.73). NHST revealed respective *p*-values of .007 and .022.

### Intervention effects

To test the effects of our intervention, we first subtracted participants' estimated ratings of the LT booklet of the estimated ratings of the HT booklets (i.e. yielding the difference score, the deviation of which from zero we tested in the previous paragraph). We then compared these difference scores at t_0_ (before our intervention) and t_1_ (after our intervention). Note that this was a comparison of two independent groups, because the participants who examined the alcohol booklets at t_0_, examined the smoking booklets at t_1_, and vice versa. The Cohen's *d*s for this comparison are positive when participants' mean ratings increased between t_0_ and t_1_, and negative when they decreased (our hypothesis is that the ratings will decrease).

Although participants did appear to decrease their ratings, these decreases were very minimal. For estimated effectiveness, Cohen's d confidence intervals ranged from -.74 to -.14 for alcohol and from -.35 to 0.21 for smoking; and for estimated risk perception, from −0.53 to 0.07 for alcohol and from −0.61 to −0.05 for smoking. None of these differences was significant according to NHST procedures, with *p*-values of .061, .739, .327 and .290, respectively.

### Comparisons with the Brown et al. Studies

Participants in the Brown et al. studies evaluated the HT booklets lower than the LT booklets, and the HT booklets had a negative effect on their risk perception. Our participants' estimations were exactly the opposite: they estimated that the HT booklets would be evaluated more positively, and that the HT booklets would cause more increases in risk perception (see Figure 3).

## Discussion

Regardless of the fact that there is much evidence about the ineffectiveness of threatening health messages, they remain widely applied in behavior change campaigns. Our study explored a possible reason: people are unable to predict the effects of threatening health messages. Most people seem convinced that threatening health messages work, and not able or willing to change this belief.

We have shown that people are unable to predict the defensive reactions to threatening health messages that occurred in the Brown and Smith and Brown and Locker studies [16,17]. Our participants predicted the emotional reactions to the booklets correctly (i.e. highly threatening messages cause higher anxiety). However, our participants did not predict the effect on estimated perceived risks and evaluations of the booklets correctly. Those were estimated to be higher after exposure to fear arousing messages, which directly contrasts with the outcomes of the Brown and Smith [16] and Brown and Locker [17] studies, which found either no effects or adverse effects of threatening health messages.

In contrast to our last hypothesis, the beliefs about the effectiveness of threatening health messages barely changed as a result of a confrontation with the empirical evidence and an explanation of the relevant dynamics. This implies that people do not readily internalize scientific evidence regarding fear appeal effectiveness, remaining convinced that threatening health messages lead to an increased risk perception. This reluctance of professionals to change when presented with evidence inconsistent with their beliefs is in line with previous results [32,33]; successfully changing professionals beliefs seems to require interventions that are a lot more sophisticated than our brief text [34].

There is one alternative explanation of this result. Perhaps participants do understand how threatening communication is processed; perhaps their error is not their assumption that threatening communications are generally effective, but their assumption that in general, efficacy levels of smokers and alcohol abusers are high. In this case, the results would be the same; participants would estimate threatening communication to be effective, while being aware that this is conditional upon high efficacy. This would still represent an estimation error, as efficacy is generally low in those exhibiting risk behaviors (see for example [35]). Although this alternative explanation would explain why our intervention did not have an effect, it is inconsistent with both anecdotal evidence from our study and evidence from another study. Some participants reported in a debriefing conversation that they simply did not believe that fear arousing messages are ineffective. They stated that intuitively, it felt right that threatening health messages influence behaviour and even clear and explicit scientific evidence could not convince them otherwise. This is in line with results found in Peters, Ruiter, & Kok [36], who found that public health professionals, advertising professionals, and decision makers often favour threatening health messages, assuming erroneously that those messages attract attention and prompt self reflection through confrontation, which ultimately leads to behaviour change.

This study suffered from a number of limitations. First, we did not check whether people actually read the provided information regarding the ineffectiveness of threatening health messages carefully enough. Note, however, that this reflects real life, where interventions aiming to educate key stakeholders as to fear appeal ineffectiveness are also not guaranteed of recipients' undivided attention. To gauge how seriously participants read the health information, we asked random participants about their experience and opinion about the given information during the debriefing, and indeed, some reported that they did not put much effort in reading the information (others indicated that they did, and some volunteered that they changed their beliefs). Another potential limitation is the fact that our sample consisted entirely of university students. This problem, the 'sophomore bias', has existed since around 1960 [37], and is by no means exclusive to the current investigation. Although the use of students as participants has recently been defended as well [38], we would like to raise an observation of Sears (1986, p. 527), namely that "the human being of strong and irrational passions […] is not that of contemporary social psychology". This reasoning would imply that if anything, using university students has worked against us: as students suffer less from "irrational passions", one would have expected these rational participants to have embraced the evidence we provided in our intervention, at least more so than a different sample.

Another aspect of the current study that strengthens its conclusions, is that in the current study, participants rated both booklets simultaneously; and the LT booklets can be considered persuasive in their own right. Thus, we can exclude the potential interpretation that the higher ratings of the HT booklets reflect the fact that participants expect threatening communication to work better than nothing; rather, the higher ratings reflect that participants expect threatening communication to have a stronger effect than another plausibly effective communication.

## Conclusion

In conclusion, people expect threatening health messages to be effective, and this belief is hard to influence by presentation of evidence to the contrary. Clearly, trying to change beliefs about the effectiveness of threatening health messages is not as easy as one might think. As shown in this study, written information with scientific evidence and theory, testifying to the ineffectiveness of threatening health messages, did not affect people’s evaluation of either the expected evaluation of threatening health messages, or the expected effect on risk perceptions. Peters et al. [36] proposed that part of the problem may be a lack of adequate alternative behaviour change methods. They suggest that when communicating with professionals, it seems fruitful to provide them with a toolbox of evidence-based behaviour change methods that promote adaptive, rather than defensive, behaviour [39-42]. The results of the current study suggest that in any case, providing scientific theory and empirical evidence does not seem to suffice to diminish the intuitive preference for threatening communications.

## Competing interests

The authors declare that they have no competing interests.

## Authors’ contributions

GH, JK, LG, KG, AH, CK, LK, IP, TS, ST and GK conceived the study, and collected and analyzed the data. GH, RR and GK designed the study. GJP analyzed the data. GH, GK, GJP and RR contributed to the interpretation of the data. GH, GJP and GK drafted the manuscript. All authors provided feedback on the manuscript and read and approved the final text.

## Pre-publication history

The pre-publication history for this paper can be accessed here:

http://www.biomedcentral.com/1471-2458/12/1011/prepub

## Supplementary Material

Additional file 1 Full disclosure of research materials, data, analyses and output. Click here for file
