# Supplementary material for: Reactions to threatening health messages
Source: BMC Public Health. 2012 Nov 21;12:1011. doi: 10.1186/1471-2458-12-1011 (PMC3575362; doi:10.1186/1471-2458-12-1011)
Supplement: Additional file 1 — Full disclosure of research materials, data, analyses and output. [file 1471-2458-12-1011-S1.zip › booklet - alcohol - HT.pdf]

Booklet B

The Menace of Alcohol

This report has been designed so that you consider the effects of excessive drinking. It has been compiled from a series of websites from around the world, all of which are reputable public health agencies, backed up by other reliable sources.

Booklet B

Taverns were first established in the 8th century. In 1688 William III licensed distilleries to make gin and between 1684 and 1727 spirit consumption rose from 0.5 to 3.5 million gallons per year. In 1734 this figure rose even further to 13.5 million and furthermore to 19 million in 1742, with licensing laws being introduced in 1729. In 1742 the government closed down all distilleries until 1759, nevertheless beer consumption during the 18th and 19th century was very high, it rose from 1.5 to 2.3 pints per person per day.

About 70% of the population drink at least occasionally, a further 10% can be regarded as ‘heavy drinkers’. Anyone at any age can have a drinking problem, meaning that their alcohol consumption is causing them harm. Alcohol slows down brain activity, affecting alertness and judgment, therefore increasing the risk of falls and accidents, however long term drinking increases the risk for many serious health problems as it affects nearly every organ in the body.

Some problems can occur after just a short period of drinking. However other problems often develop much more gradually and sometimes only become evident after years of heavy drinking. Additionally women may develop alcohol-related health problems earlier than men even when drinking less alcohol. Long term heavy drinking increases the risk for many serious health problems, like the ones described below.

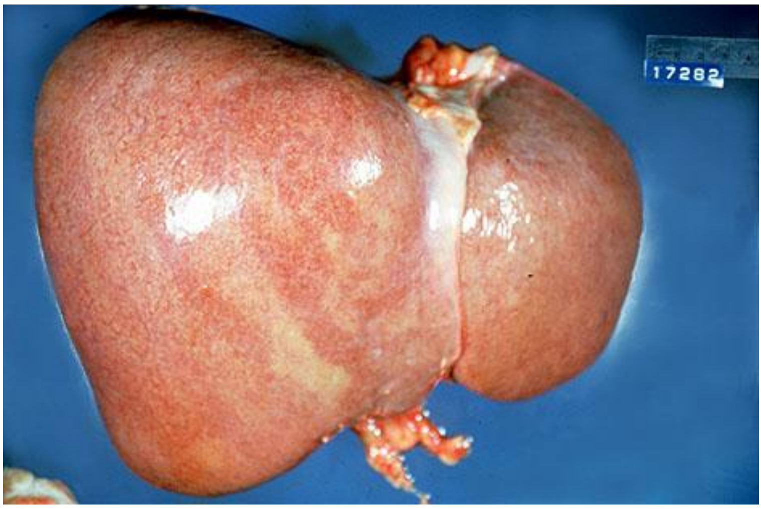

Figure 1:Enlarged liver from elderly alcoholic.

Liver Disease: Alcohol related liver disease affects thousands of people; some develop alcoholic hepatitis with symptoms inclusive of fever,

jaundice and abdominal pain, causing death if drinking does not discontinue but is reversible if drinking stops. Alcoholic cirrhosis or scarring of the liver occurs in ten to twenty percent of heavy drinkers, and if it does occur they are recommended to stop drinking immediately or they will die. Treatment is available with some severe cases requiring a transplant. However the damage is usually irreversible. A further percentage of heavy drinkers become infected with hepatitis C virus (HCV) which could cause liver cirrhosis and liver cancer.

Vascular Disease: There is an increased risk of heart disease. Heavy drinking over a long period of time increases the blood pressure and also leads to an increased risk of some kinds of stroke. Drinking more than three drinks a day has a direct toxic effect on several organs including the heart.

Cancer: Long-term heavy drinking increases the risk of some forms of cancer, particularly cancer of the esophagus, mouth, throat, and larynx, (voice box). Research has suggested that as little as one drink a day for women can raise the risk of breast cancer. Additionally drinking can increase the risk for developing cancer of the colon or rectum.

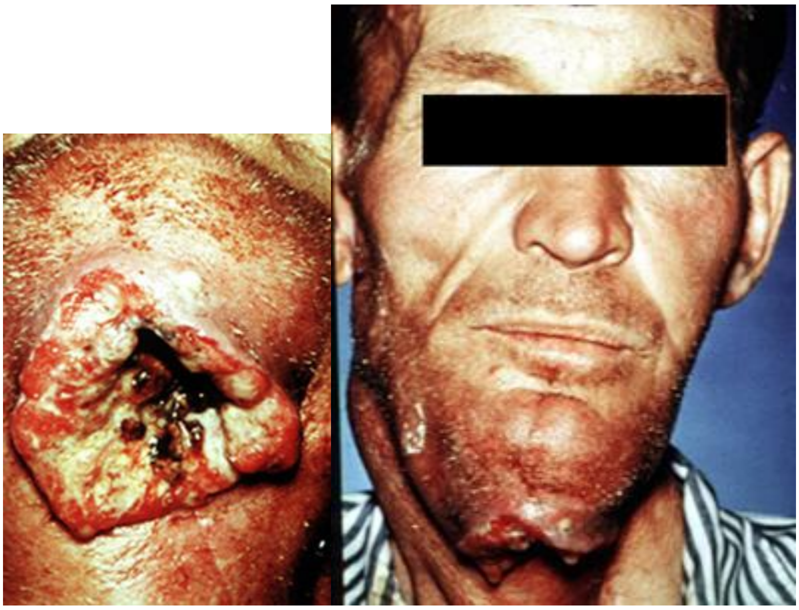

Figure 2:: Mouth and throat cancer

Pancreas: The pancreas is involved with regulating the bodies blood sugar levels with the production of insulin, furthermore it is involved in the digestion of food. Long-term heavy drinking can lead to pancreatitis (an inflammation of the pancreas), acute forms of which could cause severe

abdominal pains and be fatal. Additionally it is associated with diarrhea and weight loss.

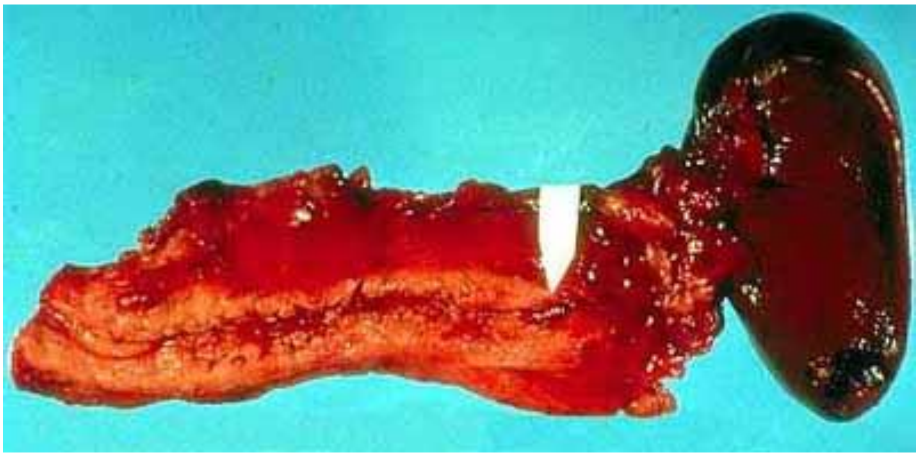

Figure 3: Chronic pancreatitis.

As well as the health problems described above, excessive drinking can also have adverse social effects.

Traffic Hazard: Alcohol remains in a woman's body for longer than a man's, and older people are less efficient at metabolizing alcohol. Due to this drink drunk-driving laws have been revised and women, especially those with a small structure and limit their intake accordingly as drink-driving could have devastating consequences. It has been suggested that 50% of drivers and 65% of pedestrians killed on the road had been drinking. Alcohol is a factor in almost 50% of deaths according to the National Institute on Alcohol Abuse and Alcoholism. At least 700 driving accidents a year involve spinal injuries to the neck, half of which involved alcohol to some effect, the alcohol in just a couple of drinks can impair one's performance enough to create a significant risk of paralytic injury. More than 4 out of 10 traffic fatalities involve alcohol and at night the traffic deaths are four times higher than during the day.

Booklet B

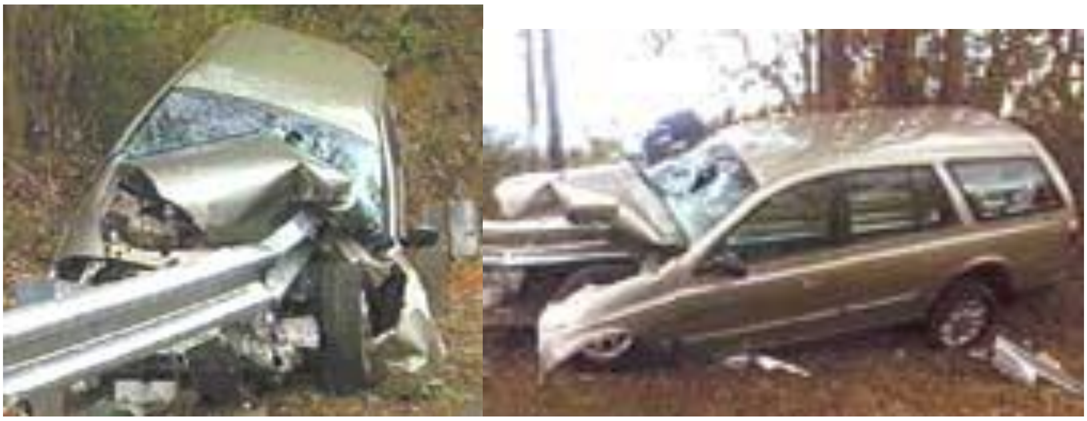

Figure 4:: Drink driving can have fatal consequences, not only to the driver but others around them too.

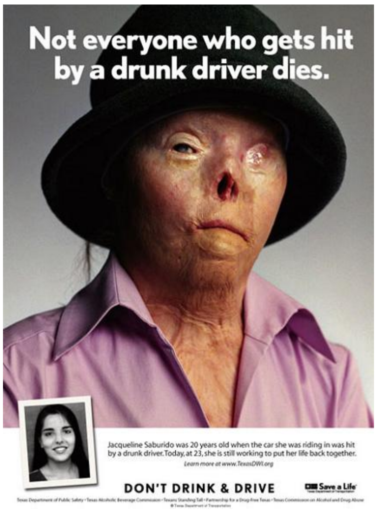

Figure 5: An advertisement against drink driving.

Antisocial Behaviour: Furthermore drinking can cause people to behave in ways they usually wouldn’t. It has been reported that 50% of all violent crimes are committed when the offender is drunk, for murders and domestic violence this figure raises to 80%.

A serious drinking epidemic occurs within the student community with every year 1,400 students between 18 and 24 dying from alcohol related injuries and many more are injured. More assault, sexual abuse, unsafe sex, academic problems, health problems, suicide attempts, drink driving, vandalism, property damage occur due to excessive drinking in students.

Booklet B

Finally this also leads to the fact that drinking at a young age, like students, is more likely to lead into a downward spiral, leading to very excessive drinking at an older age and therefore all the health problems associated with this.

If a heavy drinker reduces drinking to moderate levels, that person will improve:
The risk of Accidents,
The risk of Liver disease,
The risk of Heart disease,
The risk of Cancer,
The risk of Stroke,
The risk of Physical and Sexual victimization,
The risk of Family and Relationship Issues
Sexual functioning , and
Academic and Job Performance.

If you or someone you know has been drinking heavily there is a risk of developing serious health problems and experiencing some problems highlighted earlier. The following contacts and your general practitioner may be of assistance.

Alcohol Concern.
Waterbridge House,
32-36 Loman Street,
London.
SE1 0EE.

020 7928 7377
www.alcoholconcern.org.uk

Alcohol Problems Advisory Service.
36 Park Row,
Nottingham.
NG1 6GR

0115 948 5570
www.apas.org.uk

Alcoholics Anonymous.

www.alcoholics-anonymous.org

Booklet B
