# Supplementary material for: Reactions to threatening health messages
Source: BMC Public Health. 2012 Nov 21;12:1011. doi: 10.1186/1471-2458-12-1011 (PMC3575362; doi:10.1186/1471-2458-12-1011)
Supplement: Additional file 1 — Full disclosure of research materials, data, analyses and output. [file 1471-2458-12-1011-S1.zip › booklet - smoking - HT.pdf]

Booklet A

The Menace of Tobacco

This report is designed to stimulate you to consider giving up smoking. It is compiled from a series of websites and reports by reputable public health agencies in the UK, Canada, US and Australia. All statements are supported by reliable sources.

Booklet A

Around 2000 years ago tobacco began to be chewed and smoked. By the 1700s smoking had become a substantial international industry and smoking had become increasingly widespread. Cigarette making machines were then developed in the latter half of the 1800s. The first machines produced about 200 cigarettes per minute, but today's machines produce over 9000 per minute. Cheap mass production and the use of cigarette advertising allowed the tobacco companies to expand their markets.

During the 1920s the first medical reports linking smoking to lung cancer began to appear. Many newspaper editors refused to report these findings, as they did not want to offend tobacco companies who advertised heavily in their publications.

Figure 1: A smoker's lung showing tar in black and cancer in white.

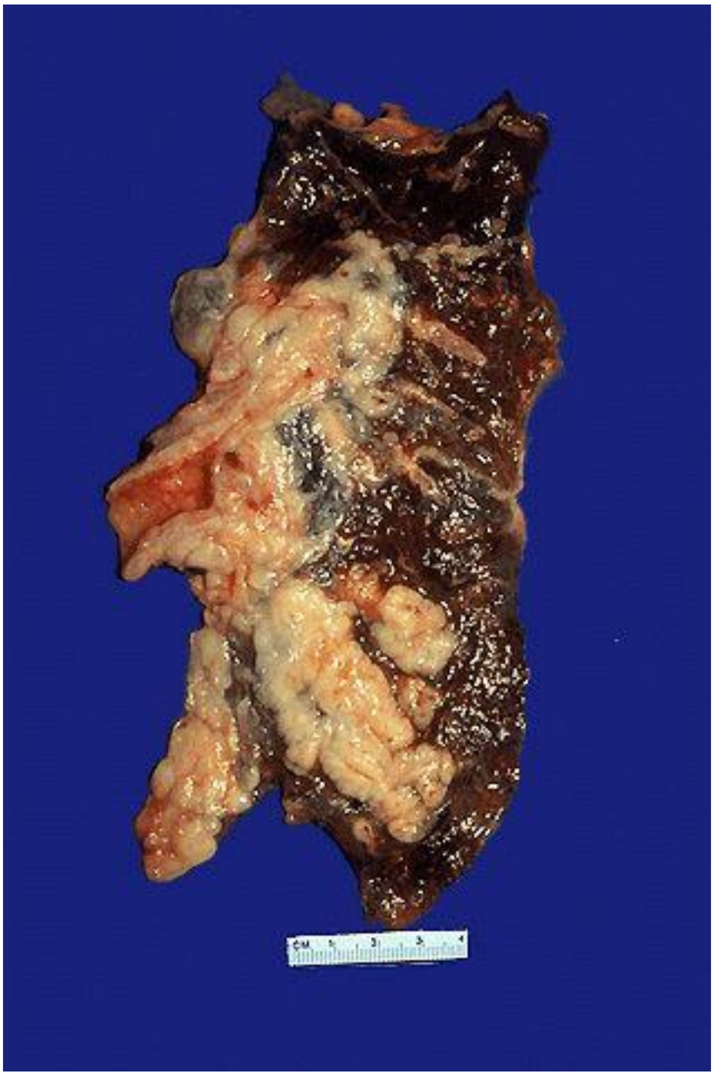

The prevalence of smoking increased dramatically during the world wars. The policy of providing free cigarettes to allied troops as a 'morale boosting' exercise contributed to this increase. In the latter half of the twentieth century there has been a rapid increase in knowledge of the health effects of both active and passive smoking. The first successful lawsuit against tobacco companies over smoking-related illness has been held.

Booklet A

Figure 2: Gangrenous foot due to poor peripheral vascular circulation.

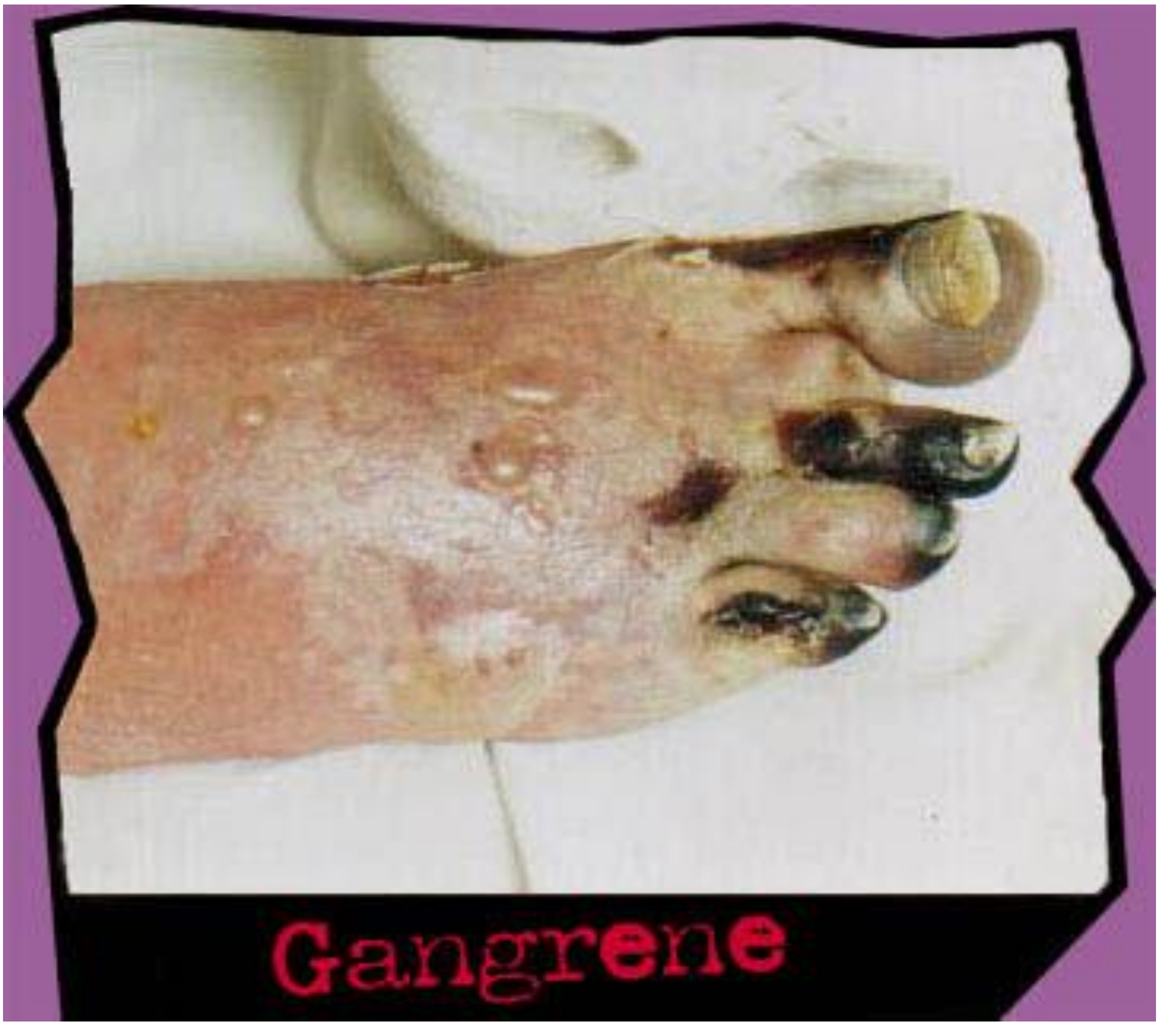

Tobacco smoking is addictive and does far greater harm than any other addictive drug. Tobacco smoke is a dangerous substance with more than 500 known poisons. Every time a smoker lights up he or she is being injured to some degree by inhaling these poisons. A two-pack-a-day smoker shortens his of her life expectancy by eight years, and even light smokers shorten their life expectancy by four years. Stopping smoking becomes extremely difficult once started and the likelihood is that individuals will continue to smoke throughout their lives.

Every day in the United Kingdom, about 450 children start smoking. By the age of 11years, one-third of children, and by 16years two-thirds of children have experimented with smoking.

About half of all regular cigarette smokers will eventually be killed by their habit. Smoking causes thirty per cent of all cancer deaths (including at least 80% of lung cancer deaths), 17% of all heart disease deaths and at least 80% of deaths from bronchitis and emphysema.

Booklet A

It is estimated that several hundred cases of lung cancer and several thousand cases of heart disease in non-smokers in the UK are caused by passive smoking. Cigarette smoking is an important cause of cancers of the lung, larynx (voice box), pharynx (throat), oesophagus, bladder, kidney and pancreas. Smoking is a cause of cancer of the nasal cavities and nasal sinuses, stomach liver, cervix and myeloid leukemia.

Figure 3: Smoker with surgically removed cancer of the lower jaw.

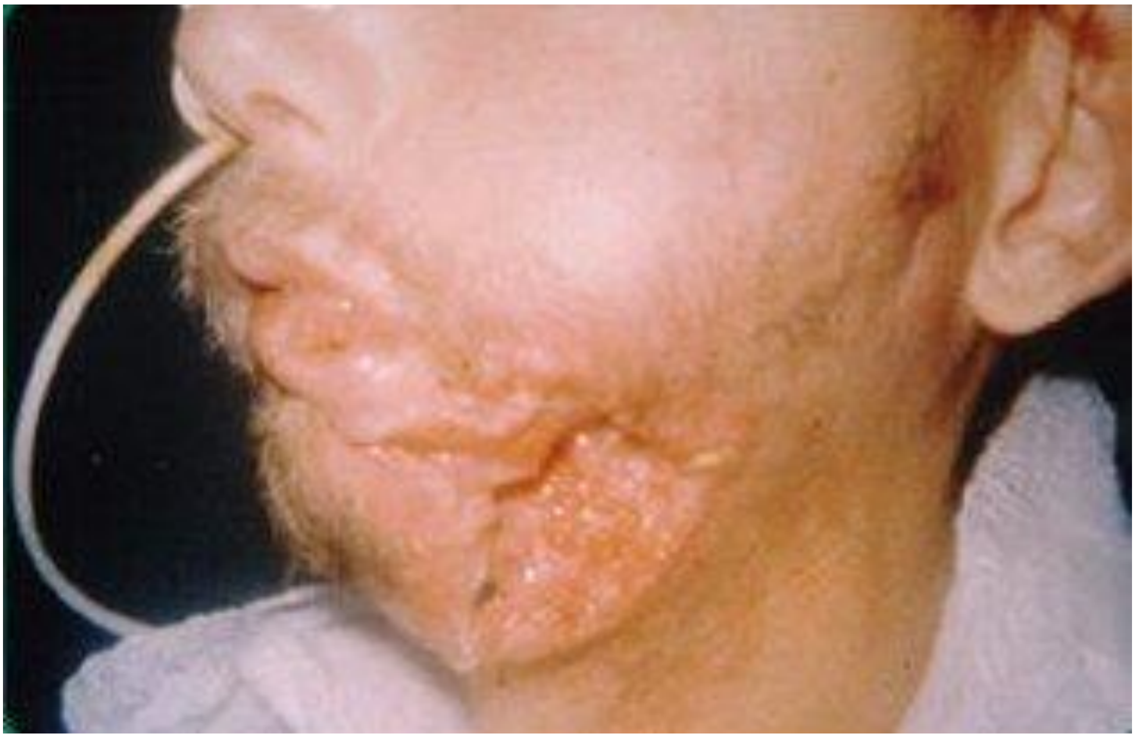

It is estimated that one in three people will develop cancer at some stage in their lives and that one in four will die from the disease. In 1995, there were 46,000 cancer deaths in the UK attributable to smoking: approximately a third of all cancer deaths. Smoking kills around six times more people in the UK than road traffic accidents (3,391), other accidents (8,933), poisoning and overdose (3157), murder and manslaughter (495), suicide (4,485), and HIV infection (180) all put together (20,641 in total - 1999 figures). Tobacco use kills around 120,000 people in the UK every year, about 330 every day - as if a plane crashed every day and killed all its passengers, around 20% of all deaths.

Tobacco companies pump millions of dollars every year into sponsoring sports events worldwide. In the United States alone, according to the Federal Trade Commission, the major domestic cigarette companies reported spending $113.6 million on sports and sporting events in 1999. In countries where direct tobacco advertising is banned by law, sponsorship of sports amounts to a cynical manipulation of national laws. Despite a federal ban on tobacco advertising on television, it is estimated that tobacco companies achieve the equivalent of more than $150 million in television advertising every year in the U.S. through their sponsorship of motor sports events.

Booklet A

When a Tobacco group sponsored the Indian World Cup Cricket in 1996, a survey showed that smoking among Indian teenagers increased five-fold. There was also marked increase in false perceptions about athletic excellence and smoking.

Figure 4: Section of smoker's lung showing emphysema.

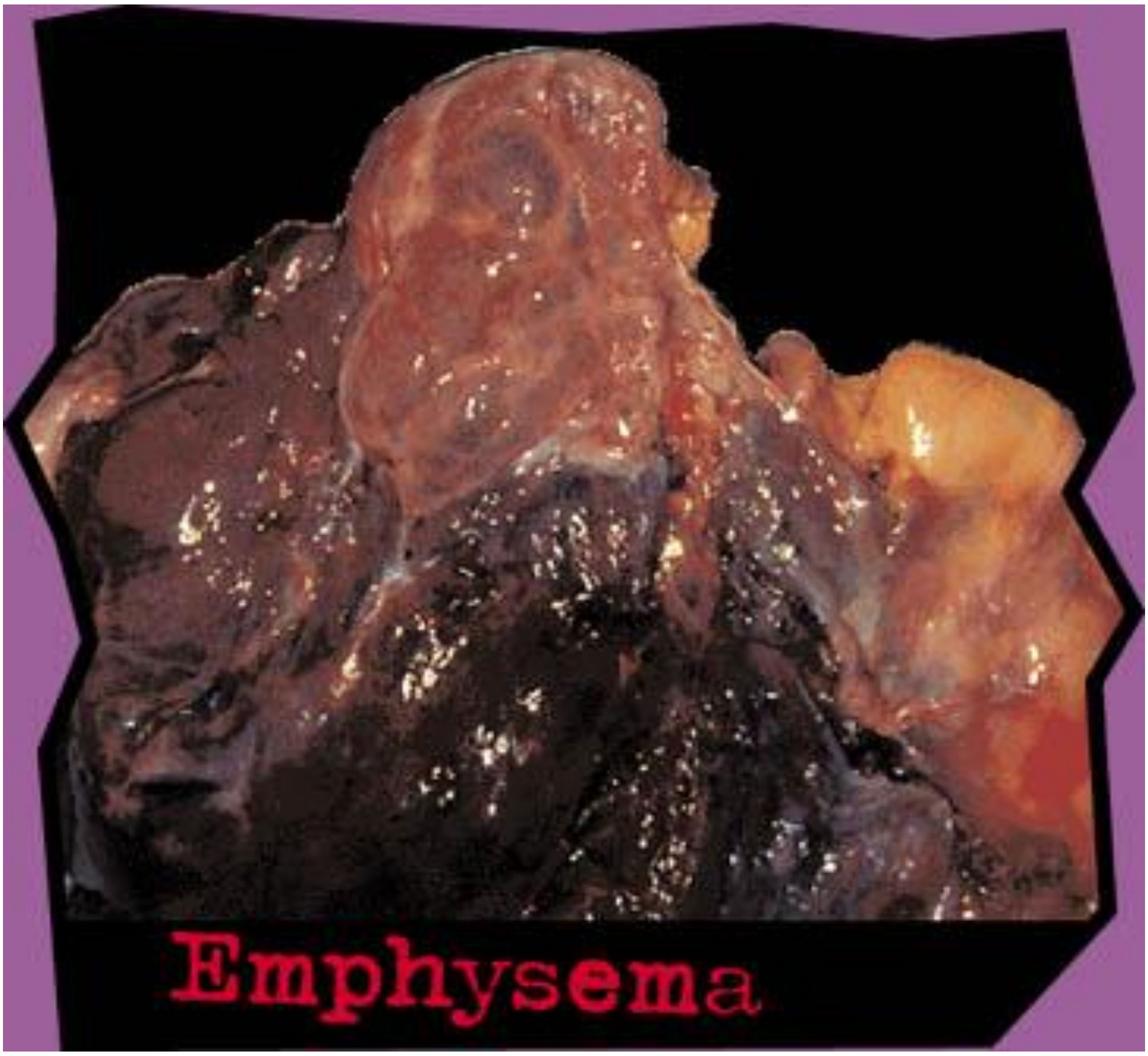

Booklet A

What Happens When You Quit

Within:	This is what happens:
20 Minutes	Blood pressure, pulse rate, and body temperature return to normal.
8 Hours	Your body starts to heal itself; the carbon monoxide level in your blood drops to normal and the oxygen level increases to normal.
24 Hours	Your chance of heart attack decreases.
48 Hours	Nerve endings start regrowing and your ability to smell and taste things is enhanced.
72 Hours	Bronchial tubes relax, lung capacity increases, and breathing becomes easier.
2 Weeks to 3 Months	Circulation improves, walking becomes easier, and lung function increases up to 30%.
1-9 Months	Cilia reactivate in the lungs, increasing their ability to handle mucous, clean the lungs and reduce infection. Coughing, sinus congestion, fatigue and shortness of breath decrease and your body's overall energy level increases.
5 Years	The lung cancer death rate for the average smoker decreases from 137 per 100,000 people to 72 per 100,000 people (almost a 50 percent reduction).
10 Years	The lung cancer death rate for the average smoker drops to almost the rate of nonsmokers (12 per 100,000 people). The risk for other cancers (mouth: larynx, esophagus, bladder, kidney, pancreas) also decreases.
